# P-1463. Ceftaroline Versus Vancomycin for the Treatment of Complicated Skin and Soft Tissue Infections (cSSTIs): A Retrospective Cohort Study

**DOI:** 10.1093/ofid/ofae631.1635

**Published:** 2025-01-29

**Authors:** Ali Althubyani, Dana Holger, Eka Beriashvili

**Affiliations:** Nova Southeastern University, Davie, Florida; Nova Southeastern University, Davie, Florida; Memorial Healthcare System, Hollywood, Florida

## Abstract

**Background:**

Complicated skin and soft tissue infections (cSSTIs) caused by resistant pathogens like methicillin-resistant *Staphylococcus aureus* (MRSA) require effective treatment. Vancomycin is the standard-of-care for MRSA-associated cSSTIs, but its efficacy is questioned due to poor tissue penetration. Ceftaroline, an anti-MRSA cephalosporin with improved tissue penetration, may offer better outcomes. This real-world study compares the effectiveness of ceftaroline and vancomycin in treating cSSTIs.

**Figure:**
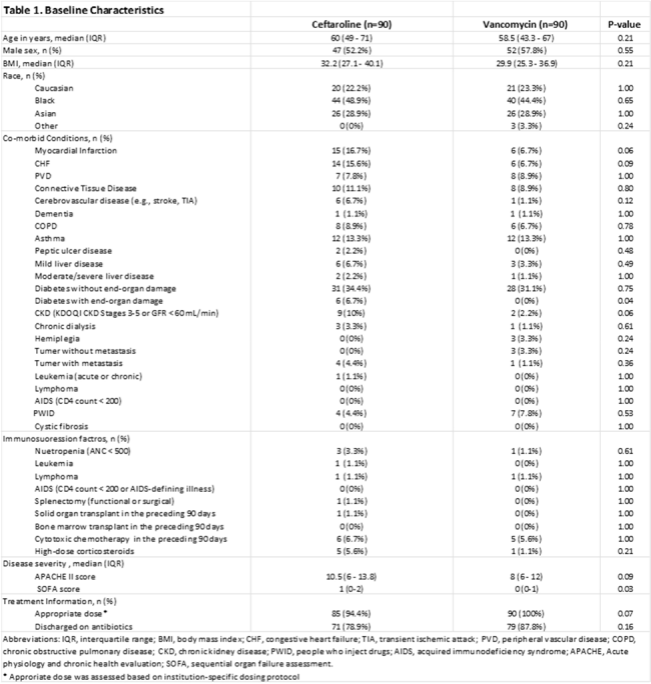

**Methods:**

A multicenter retrospective cohort study was conducted in five Memorial Health System hospitals from 2019-2023. Here we present an interim analysis of 90 patients per arm. Included were adults diagnosed with cSSTIs who received ≥48 hours of ceftaroline or vancomycin treatment and were discharged alive. Exclusions were patients with any concurrent infection at initial diagnosis, infections requiring prolonged therapy, combination therapy, pregnancy, or incarceration. The primary outcome was clinical cure rate. Secondary outcomes were hospital length of stay (LOS), 30-day readmission, 30-day recurrence, time to oral antibiotic switch, and nephrotoxicity. Multivariable logistic regression was used to assess the independent association between ceftaroline therapy and clinical cure, adjusting for confounders.

**Figure:**
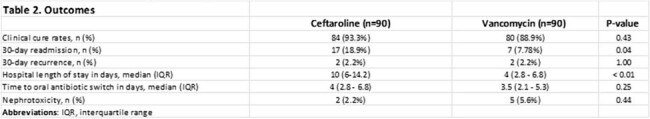

**Results:**

Overall, 180 patients were included. For the ceftaroline and vancomycin groups, median (IQR) APACHE scores were 10.5 (6-13.8) and 8 (6-12) (p=0.09), and SOFA scores were 1 (0-2) and 0 (0-1) (p=0.03), respectively. Clinical cure rates were 93.3% for ceftaroline and 88.9% for vancomycin (p=0.43). Multivariate analysis showed ceftaroline is independently associated with clinical cure (p=0.04). For vancomycin and ceftaroline, median (IQR) LOS was 4 days (2-6) versus 10 days (6-14) (p< 0.01), and 30-day readmission rates were 7.8% and 18.9%, respectively (p=0.04). No significant differences in other outcomes were noted.

**Figure:**
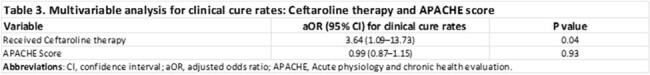

**Conclusion:**

Interim analysis shows ceftaroline is associated with numerically higher clinical cure rates than vancomycin, despite higher disease severity in the ceftaroline group. Ceftaroline is an independent predictor of clinical cure. Further investigation is needed to compare the efficacy and safety of both treatments.

**Disclosures:**

**All Authors**: No reported disclosures

